# A precautionary public health protection strategy for the possible risk of childhood leukaemia from exposure to power frequency magnetic fields

**DOI:** 10.1186/1471-2458-10-673

**Published:** 2010-11-05

**Authors:** Myron Maslanyj, Tracy Lightfoot, Joachim Schüz, Zenon Sienkiewicz, Alastair McKinlay

**Affiliations:** 1 Health Protection Agency, Chilton, Didcot, Oxfordshire, OX11 0RQ, UK; 2 Epidemiology & Genetics Unit, Department of Health Sciences, Seebohm Rowntree Building, University of York, York YO10 5DD, UK; 3 Institute of Cancer Epidemiology, Danish Cancer Society, Strandboulevarden 49, 2100 Copenhagen, Denmark; 4 International Agency for Research on Cancer (IARC), 150 Cours Albert Thomas, 69372 Lyon Cedex 08, France

## Abstract

**Background:**

Epidemiological evidence showing a consistent association between the risk of childhood leukaemia and exposure to power frequency magnetic fields has been accumulating. This debate considers the additional precautionary intervention needed to manage this risk, when it exceeds the protection afforded by the exposure guidelines as recommended by the International Commission on Non-Ionizing Radiation Protection.

**Methods:**

The Bradford-Hill Criteria are guidelines for evaluating the scientific evidence that low frequency magnetic fields cause childhood leukaemia. The criteria are used for assessing the strength of scientific evidence and here have been applied to considering the strength of evidence that exposures to extremely low frequency magnetic fields may increase the risk of childhood leukaemia. The applicability of precaution is considered using the risk management framework outlined in a European Commission (EC) communication on the Precautionary Principle. That communication advises that measures should be proportionate, non-discriminatory, consistent with similar measures already taken, based on an examination of the benefits and costs of action and inaction, and subject to review in the light of new scientific findings.

**Results:**

The main evidence for a risk is an epidemiological association observed in several studies and meta-analyses; however, the number of highly exposed children is small and the association could be due to a combination of selection bias, confounding and chance. Corroborating experimental evidence is limited insofar as there is no clear indication of harm at the field levels implicated; however, the aetiology of childhood leukaemia is poorly understood. Taking a precautionary approach suggests that low-cost intervention to reduce exposure is appropriate. This assumes that if the risk is real, its impact is likely to be small. It also recognises the consequential cost of any major intervention. The recommendation is controversial in that other interpretations of the data are possible, and low-cost intervention may not fully alleviate the risk.

**Conclusions:**

The debate shows how the EC risk management framework can be used to apply the Precautionary Principle to small and uncertain public health risks. However, despite the need for evidence-based policy making, many of the decisions remain value driven and therefore subjective.

## Background

Leukaemia is the most common type of childhood cancer, accounting for 30% of all cancers diagnosed in children younger than 15 years [1,2]. Within this population, acute lymphoblastic leukaemia (ALL) occurs approximately five times more frequently than acute myeloid leukaemia (AML), contributing to about 80% of all childhood leukaemia diagnoses [2]. Power frequency electric and magnetic fields are a ubiquitous feature of modern life, and encountered wherever electricity is used. Common sources include overhead power lines, local electricity distribution networks and substations, as well as wiring circuits and electrical appliances [3]. Since 1979, more than 20 epidemiological studies have investigated the possibility that exposure to power frequency magnetic fields may be a risk factor in the development of childhood leukaemia. A number of the studies have been pooled in four meta-analyses which point to an approximate doubling of risk at average residential levels of 0.3-0.4 microtesla (μT) [4-7].

Exposure guidelines such as those published by the International Commission on Non-Ionizing Radiation Protection (ICNIRP) [8] are used in many countries to protect members of the public from the harmful effects of power frequency electric and magnetic fields. In the European Union, there is a Council Recommendation on limiting exposure of the general public which looks to compliance with the ICNIRP guidelines [9]. The guidelines set restrictions to prevent what are considered to be the known adverse effects of exposure - those relating to electric fields and currents in tissues of the central nervous system. The guidelines are cautious in that they use reduction factors to allow for various sources of uncertainty and the potential sensitivities of certain population groups. Nevertheless the guideline reference level of 100 μT for power frequency magnetic fields is much higher than the average environmental level implicated in the epidemiological studies. The threat of harm suggested by the epidemiological studies is seen as a possible justification for invoking additional precautionary measures over and above the protection afforded by the exposure guidelines.

The Precautionary Principle is an increasingly influential aspect of modern policy making, challenging regulators to take steps to protect against potential harms, even if causal chains are uncertain [10-12]. There has been much discussion of the principle in abstract and general terms, but its meaning and role in the practical management of minor and uncertain risks is ambiguous and controversial. The European Commission (EC) has taken a leading role in fostering discussion on the application of the Precautionary Principle, mainly through a communication which establishes guidelines for applying it [13].

This paper considers the application of precaution to address the possible risk of childhood leukaemia from exposure to power frequency magnetic fields. The Bradford-Hill Criteria are used to evaluate the scientific evidence and precaution is considered within the risk management framework of the EC communication on the Precautionary Principle.

## Methods

The first part of the evaluation uses the Bradford-Hill Criteria [14] to examine the strength of evidence that suggests power frequency magnetic fields cause childhood leukaemia. The criteria are a useful guide to evaluating whether or not an observed association reflects causality. The pros and cons with respect to the question of association or causation are considered, and areas of uncertainty are identified.

The second part of the evaluation considers the applicability of precaution within the risk management framework outlined in the EC communication on the Precautionary Principle [13]. The framework requires measures to be proportionate, non-discriminatory, consistent with similar measures already taken, based on an examination of the benefits and costs of action and inaction, and subject to review in the light of new scientific findings.

## Results

### Science-based risk assessment

Table 1 summarises the evidence suggesting that power frequency magnetic fields may cause childhood leukaemia with reference to the Bradford-Hill Criteria [14]. For comparison, the evidence for ionising radiation, a well-known carcinogen, causing leukaemia, is also summarised in the table. In general, the evidence suggesting that power frequency magnetic fields cause childhood leukaemia is considered to be relatively weak, and the main categories that fall short are strength of association, dose-response relationship, biological plausibility and coherence, and analogy.

**Table 1:** Summary evidence in terms of Bradford-Hill Criteria [14] for power frequency magnetic fields causing childhood leukaemia.

Bradford-Hill Criterion	Power frequency magnetic fields	Ionising radiation
*Strength of Association*	Pooled studies suggest a statistically significant doubling of risk above 0.3-0.4 uT. The strength of association is considered to be weak and only a small proportion of cases are attributable to high exposure.	Statistically significant raised risks of leukaemia are observed with increasing exposure to ionising radiation. Risk estimates are extrapolated from epidemiological data at higher doses using a linear no-threshold exposure response model.

*Consistency*	The association is observed almost exclusively in childhood case-control studies.	The association is observed in two different situations: first, studies of Japanese atomic bomb survivors irradiated as children, and second, studies of childhood cancer and antenatal exposure of the foetus to diagnostic X-rays.

*Specificity*	The association seems to be restricted to leukaemia, athough other childhood cancers have been investigated less frequently and less rigorously.	Studies have demonstrated that a number of different cancers are associated with exposure to ionising radiation.

*Temporality*	In ALL, the most common type of childhood leukaemia, the disease occurs relatively rapidly after exposure, normally in the third or fourth year of life.	In many of the cancers associated with ionising radiation, exposures can precede lesions by as much as several decades.

*Dose response relationship*	There are too few data, even after pooling, to identify the shape of a possible dose-response relationship. Threshold exposure response models have been suggested although data are also compatible with other trends.	A linear-quadratic dose response relationship is found between childhood leukaemia and ionising radiation exposure in A-bomb survivor studies, except at the highest levels of exposure. The shape of the dose-response curve is uncertain at low doses.

*Biological plausibility*	A number of mechanisms have been proposed for the interaction of magnetic fields with the human body, but it is unclear how these might affect the processes that lead to disease, particularly at the low levels identified in the epidemiological investigations. *In vitro *and *in vivo *experiments fail to show a consistent effect that might explain the development of childhood leukaemia.	There is a good mechanistic basis for suggesting ionising radiation causes leukaemia, involving direct damage to DNA. There are also other processes that have the potential to modify the simple model. There is abundant *in vitro *and *in vivo *evidence to support the carcinogenic effect of ionising radiation.

*Biological coherence*	The cause of childhood leukaemia is complex and not well enough understood to make an assessment.	The observed associations are consistent with what is known about the carcinogenic effects of ionisation radiation.

*Experiment (reversibility)*	Evidence that removing the exposure reduces disease would be difficult to ascertain because of the small fraction affected.	Evidence is difficult to ascertain.

*Analogy*	No analogies in adjacent parts of the electromagnetic spectrum.	A leukaemogenic effect is consistent with what is known about ionising radiation causing a range of cancers.

For comparison purposes, the same criteria are considered for ionising radiation causing leukaemia

The conclusion is in accord with the findings of a number of authoritative bodies that have reviewed the scientific evidence and acknowledged the possibility of a risk, including the independent Advisory Group on Non-ionising Radiation [15-17], ICNIRP [18], the International Agency for Research on Cancer (IARC) [19] and the National Radiological Protection Board (now the Health Protection Agency) [20]. More recent reviews which continue to acknowledge the possibility of a risk include those by the Health Council of the Netherlands [21,22], the Swedish Radiation Protection Institute [23,24], the World Health Organization (WHO) [25], the Danish Cancer Society [26], and the EU Scientific Committee on Emerging and Newly Identified Health Risks (SCENIHR) [27,28].

On the basis of the epidemiological evidence, IARC classified power frequency magnetic fields as a possible human carcinogen (Group 2B) [19,29]. The IARC evaluation concluded that in humans there was limited evidence for carcinogenicity of extremely low frequency magnetic fields in relation to childhood leukaemia; inadequate evidence for the carcinogenicity of extremely low frequency magnetic fields in relation to all other cancers; and inadequate evidence in experimental animals for the carcinogenicity of extremely low frequency magnetic fields [19].

**Figure 1:**
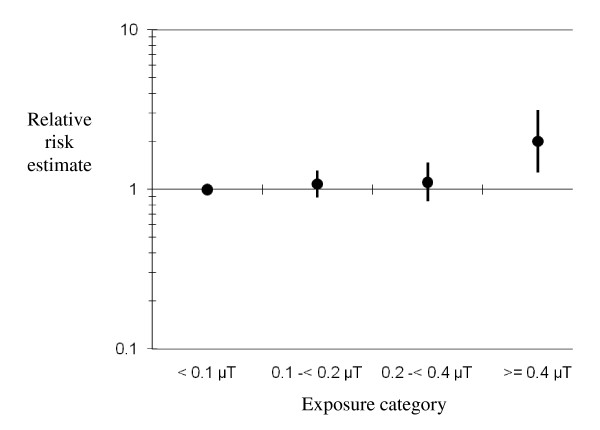
**Pooled relative risk estimates from the Ahlbom *et al *meta-analysis on residential magnetic fields **[4].

The epidemiological evidence for the association is illustrated in Figure 1 and Table 2, using the analysis of Ahlbom *et al *[4]. The Ahlbom *et al *study was based on the geometric mean magnetic field level in nine studies and suggested that exposure to power frequency magnetic fields in the home above an average of 0.4 μT was associated with a doubling of the risk of leukaemia in children less than 15 years of age. In a separate, but similar, pooled analysis [5], the arithmetic mean was used to examine the association in twelve studies and a similar level of risk was observed at a slightly lower cut-point of 0.3 μT. The advantage of using the results from the pooled analyses for risk assessment is their larger numbers and the harmonisation of the statistical approach to analyse the data, particularly the choice of cut-off points to categorize exposure [30]. Looking at the individual studies is of little use to evaluate consistency, because individual studies have only few, if any, subjects in the exposure categories that demonstrated an association in the pooled analyses. This is also why the magnetic field value used in the individual studies to define "high exposure" is highly variable, reaching from 0.1 to 0.5 μT. This is illustrated by the studies pooled by Ahlbom *et al *[4] and shown in Table 2; three of the nine studies had no cases and/or controls in the high exposure category, while the overall results were mainly driven by one single US study [31], providing 36% of all exposed leukaemia cases.

**Table 2:** Power frequency magnetic fields and the risk of childhood leukaemia - results from nine studies included in the pooled analysis of Ahlbom *et al *[4]

		Leukaemia cases	
	Odds Ratio (95% CI) ≥ 0.4 μT vs. < 0.1 μT	Observed ≥ 0.4 μT	Expected ≥ 0.4 μT

Canada	1.55 (0.65 - 3.68)	13	10

USA	3.44 (1.24 - 9.54)	17	5

UK	1.00 (0.30 - 3.37)	4	4

Norway	0 cases, 10 controls	0	3

Germany	2.00 (0.26-15.17)	5	2

Sweden	3.74 (1.23 - 11.4)	5	2

Finland	6.21 (0.68 - 56.9)	1	0

Denmark	2 cases, 0 controls	2	0

New Zealand	0 cases, 0 controls	0	0

Total	2.00 (1.27 - 3.13)	47	26

More recent studies continue to confirm an association [32]. A large case-control study conducted in England and Wales found higher rates of childhood leukaemia among those born within 600 m of a high voltage power line compared with those born further away [33], although magnetic fields are unlikely to be the cause of the whole increase [34]. In addition studies examining survival or particularly susceptible groups [35-37] support the possibility of a risk. A pooled analysis investigating whether exposure at night revealed a stronger association confirmed an overall doubling in risk, but not a higher risk with increased exposure at night. The main rationale for focusing on night-time exposure was that because the child is more permanently at the place where the measurement was taken, dilution of the association by exposure misclassification might be reduced [6]. A recent pooled analysis of studies conducted after the publication of the previous pooled analyses by Ahlbom *et al *[4] and Greenland *et al *[5] combined seven new studies and observed pooled effect estimates compatible with the previous studies, although slightly weaker [7]. Interestingly, a recent pooled analysis of epidemiological studies on childhood brain tumours, several of them conducted in connection with the childhood leukaemia studies i.e. with identical methodology, showed a pooled effect estimate of 1.14 (95% CI: 0.61, 2.13) at magnetic field levels ≥ 0.4 μT, suggesting little evidence for an association between magnetic field exposure and risk of childhood brain tumours [38].

### Scientific uncertainty

As yet, there is no clear explanation for the observed association; it could arise if power frequency magnetic fields have a causal role in the development of the disease or, alternatively, it could arise as a result of a statistical artefact reflecting selection bias, confounding or chance [28]. The probability is that selection bias alone is not sufficient to explain the entire association, although it is likely to have led to an over-estimation of the observed association. This over-estimation is due to a deficit in participation of lower socioeconomic status controls, a group that has been shown to have a higher likelihood of living in apartments with elevated magnetic field levels. The resulting under-representation of control families with expected higher magnetic field exposure has spuriously strengthened the association, e.g., for the German study it was estimated that 66% of the association was likely to be attributable to selection bias [26,29]. Confounding by a factor that is related both to magnetic fields and the risk of leukaemia appears to be unlikely, as such a factor would need to be a rather strong risk factor for leukaemia even when virtually perfectly correlated with magnetic field levels, and such a factor is not known [39]. However, since the observed increased risk is based on relatively small numbers of exposed children, a combination of selection bias, confounding and chance cannot be ruled out as an explanation for the observed association [29].

The evidence for a causal relationship would be strengthened considerably if experimental studies were to demonstrate that magnetic fields affect biological systems at the exposure levels implicated in the epidemiological studies. The various mechanisms by which magnetic fields might interact with the body have been considered by a WHO Task Group [25]. However, most are only likely to affect biological processes at very high field levels, far above those identified in the epidemiological studies. There is no consistent evidence from laboratory studies, both in vitro and in vivo, that low level magnetic fields can damage DNA, or induce any type of cancer [25].

In addition to investigating the possible direct acting carcinogenic properties of magnetic fields, indirect roles in leukemogenesis have also been suggested, including mechanistic links related to corona ions from power lines [40-42], suppression of nocturnal production of the oncostatic hormone melatonin by magnetic fields [43] and that the increased occurrence of contact currents in residences with higher magnetic fields leads to higher bone marrow doses of induced currents as well as magnetic fields via contact with metallic water fixtures during bathing of the child [44]. However, these hypotheses are speculative and any effects are considered to be small or unknown [45,46,25].

It cannot be excluded nevertheless that the lack of effect seen overall in the experimental laboratory studies could in part be due to lack of appropriate models for the complex processes that lead to the development of childhood leukaemia. There is, therefore, perhaps the need for new and/or refined models to be developed and tested in order to conclusively demonstrate that exposure to magnetic fields at the relevant environmental levels neither induces molecular and genetic changes associated with leukaemia initiation, nor drives disease progression.

The absence of supporting experimental evidence also needs to be considered in the context of how little is known about the development of the disease. The causes of most types of leukaemia are largely unknown [1,2,25]. Ionising radiation is a recognised risk factor [47]. Whilst some data suggest links with solvents, pesticides, tobacco smoke and certain dietary agents, the evidence is generally weak. Even where associations are observed, these would explain only a small proportion of the disease cases, leaving the majority with unexplained aetiology [48]. The weak associations identified for a number of hypothesised risk factors imply that multiple pathways may be involved in disease development, and as with other multifactorial diseases, gene interactions with environmental factors may also modulate disease risk [48-56].

The potential of power frequency magnetic fields to cause diseases other than childhood leukaemia has received less attention [19,25]. SCENIHR noted in its 2009 report to the European Commission [27], that while a number of health effects had at first appeared to be associated with extremely low frequency (ELF) fields; many of these possibilities have been dismissed based on information from later research. This holds, for example, for cardiovascular disease. However, for some diseases SCENIHR concluded that it still remains open as to whether there is a link to ELF exposure. This was true for neurodegenerative diseases in particular, such as amyotrophic lateral sclerosis (ALS) and Alzheimer's disease [57,58]. Findings from studies published after the SCENIHR report, including one on railway workers [59] and one on people residing in the proximity of power lines [60], support the possibility that Alzheimer's disease might be linked to exposure to ELF fields.

### Consideration of precaution within the EC risk management framework

#### 1) Proportionality

According to the EC communication, the measures based on the Precautionary Principle must not be disproportionate to the desired level of protection and must not aim at zero risk. This reflects the Principle of Proportionality used in EU law, which dictates that measures implemented through Community provisions must be appropriate for attaining the objective pursued and must not go beyond what is necessary to achieve it, thus preventing the unreasonable use of precaution [61].

Here, in the context of childhood leukaemia and magnetic fields, the scientific uncertainty may be sufficient to trigger the application of precaution, but the likely magnitude of the risk would argue against high-cost intervention to reduce exposure. For example, cancer in children is rare, and the cumulative risk of developing leukaemia before the age of 15 in the UK equates to approximately 1:1,500 [62]. At the same time, advances in treatment mean that over 70% of children survive for over 10 years [62]. The pooled epidemiological studies [4-7] use threshold models which suggest that there is an approximate doubling of leukaemia risk for children exposed at levels above 0.3-0.4 μT. In the UK this is equivalent to an increase in the annual risk of the disease in children from 1 in 20,000 to 1 in 10,000, and an increase in cumulative risk up to the age of 15 years from 1 in 1,500 to 1 in 750. A WHO task group estimated that between 100 and 2,400 childhood cases per year worldwide could be attributable to magnetic field exposure above 0.3 μT [25]. If the risk is real, this represents 0.2 - 4.9% of the total annual number of leukaemia cases worldwide [25]. In the UK, exposures at this level are relatively rare [63] and central estimates suggest that magnetic field exposure from all sources combined would contribute up to about 5 of the 500 cases which occur each year, and only a proportion of these would be attributable to high voltage power lines [3,64]. Another study which focused on proximity to high voltage power lines has put this figure as high as 25, on the assumption that the risk extends out to 600 m from a line [33], much greater than the distance where magnetic fields from the line would be elevated [33,34,65]. Thus, even assuming a causal relationship, the disease burden attributable to exposure would appear to be small.

#### 2) Non-discrimination

Much of the discussion has focused on reducing the exposure from high voltage power lines, either by restricting building of homes in the vicinity of lines or *vice versa*. However, recent evidence in the UK suggests that restricting precaution to high voltage power lines may be discriminatory, in that many low-voltage sources are also associated with high exposure [3,64]. In the UK, low voltage sources associated with the final electricity supply are estimated to account for 77% of exposures above 0.2 μT, and 57% of those above 0.4 μT [3]. Most of these exposures are linked to net currents in circuits inside and/or around the home. The high-voltage sources, including the power lines that are the focus of public concern, account for 23% of the exposures above 0.2 μT, and 43% of those above 0.4 μT [3,64]. Thus if precautionary measures are deemed to be necessary, then action should be taken for both these sources of risk.

#### 3) Consistency

The consistency criterion requires that the measures should be of comparable scope and nature to those already taken in equivalent areas in which all the scientific data are available. The criterion is difficult to evaluate because there are no obvious parallels in adjacent parts of the electromagnetic spectrum and the causes of the disease remain largely unknown. In relation to ionising radiation, where carcinogenic effects are relatively well established, the as low as reasonably achievable (ALARA) approach is taken which assumes a linear no-threshold exposure-response model. In relation to chemical pollutants, the converse is often true i.e. there may be good experimental evidence suggesting the possibility of harm but the evidence from human health studies is more difficult to establish. Thus the consistency criterion is difficult to apply and does not add much to clarify the issue.

#### 4) Cost-benefit

**Figure 2:**
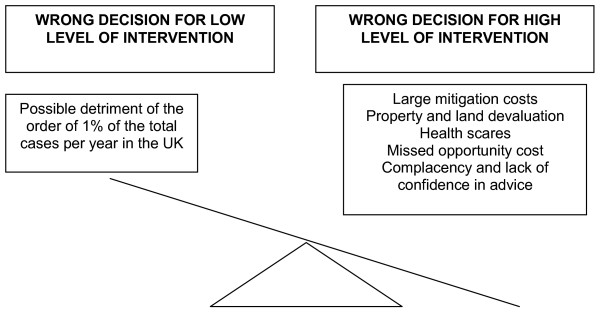
**The costs of wrong decisions for high and low level interventions to reduce exposure**.

The consideration of cost-benefit is an important criterion to adhere to in evaluating a particular intervention. Its scope in the EC communication is much broader than a purely economic cost-benefit assessment, stating it includes non-economic considerations such as efficacy of possible options and their acceptability to the public. Figure 2 summarises what is considered to be the situation for childhood leukaemia and magnetic fields. Different strengths of evidence are required in different situations depending on the outcome, and this is essentially dependent on the likely costs of being wrong in acting, or not acting, to eliminate or reduce exposure [14,61]. Bradford-Hill stressed that in real life, consideration should be given to what flows from a decision [14]. Here we suggest that relatively high economic and societal costs would be incurred to sustain what appears to be a small and uncertain health benefit. Thus it follows that only inexpensive actions can be justified.

#### 5) Examination of scientific developments

Implicit in the application of the Precautionary Principle is a commitment to review the arrangements and to carry out research aimed at understanding the underlying issue [12,66]. Analogy has been drawn between the results of epidemiological studies and the preliminary screening tests that are used in healthcare and medicine [67]. The initial screening tests are not usually sufficient in themselves to identify or manage a risk, as they are dominated by a large proportion of false positives. Such circumstances call for the gathering of sequential evidence, ideally from more than one source, and targeting of higher risk groups for screening. In the present context, this may translate to parallel studies on susceptible subgroups in relation to magnetic fields and childhood leukaemia, and more experimental research to establish how magnetic fields might influence the complex biological processes that lead to the disease.

## Discussion

The strengths and weaknesses of the Precautionary Principle as a risk management strategy have been reviewed elsewhere [10-12,66-69]. It has been suggested that the principle is good for public health because it promotes the search for safer technologies, encourages openness in policy and stimulates re-evaluation of methods in public health science [12]. Substantial action would normally be justifiable in circumstances where there were likely to be severe consequences from failing to detect a rare hazard. On the downside, interventions to reduce exposure can be costly and complacency or lack of public confidence may arise, especially if there turns out to be no risk [67].

Issues surrounding the application of precautionary intervention to public health risks have been elaborated by various authors [12,66,67]. For instance, Hrudey and Leiss contrasted two drinking water incidents [67]; the first was in 1998 in Sydney, Australia when residents were advised to boil water on the basis of erroneous monitoring results which produced a false positive error. This resulted in several million dollars being spent on an incident where public health had apparently not been endangered; such responses may undermine public confidence and cause complacency at times when precautionary measures are truly needed. The second example in Walkerton, Ontario, Canada, was when warnings ignored by operators and regulators resulted in the outbreak of a fatal waterborne disease; a case of a false negative error [67].

Early preventative action has been recommended by Gee [61] to limit exposure to various environmental toxicants in order to prevent reproductive or developmental harm. Gee noted that the actual evidence linking particular disorders with specific exposures was not very strong, but suggested that this was only to be expected given the limitations of applying current scientific methods to complex multi-causal and often reciprocal systems and disease processes. Another recent example, this time from the UK, was the use of a precautionary approach to manage the possible health risks associated with the use of mobile phones [70].

The evaluation presented in this debate is consistent with other studies which suggest that precautionary action is warranted [20,25]. In 2004, the UK National Radiological Protection Board, now the Health Protection Agency, concluded that it was important to consider the need for additional precautionary measures over and above the protection afforded by the ICNIRP guidelines [20]. In 2007, a WHO Task Group concluded that the consistent epidemiological evidence for an increased risk of childhood leukaemia associated with chronic low intensity magnetic field exposure was sufficient to warrant precautionary action [25]. However, given both the weakness of the evidence for a link and the limited impact on public health, the benefits of exposure reduction are unclear, and therefore, any costs to reduce exposure should be very low [25].

The main conclusion of this evaluation, namely only low-cost interventions should be pursued at this time, is critically dependent on the assumption that if the risk is real, its impact is likely be small. The Bradford-Hill Criteria have been used as the basis for the evaluation; however, it is also acknowledged that very few causal agents meet all these criteria, and whilst support of the criteria can be robust evidence for a causal association, the complex and multi-causal nature of biological interactions means that the converse is not necessarily true [61]. The evaluation is also somewhat limited in that a comprehensive public health assessment should ideally take into account a wide range of chemical, biological and physical risk factors.

The small impact assumption is based on applying a threshold model to the data; however, the precise relationship of the exposure-response model is unknown, and although the risk becomes detectable at around 0.3-0.4 μT, the observed data are consistent with trend models that are nearly flat, or curves that rise and then fall, or even curves that rise exponentially [5,6,71]. If a linear no-threshold model is postulated, the number of attributable cases becomes greater. Study biases and uncertainties in the exposure distributions could also make the attributable fraction somewhat larger [72]. There is also the possibility of susceptible subgroups and other disease end-points.

The interpretation of 'low-cost' is inherently subjective. It is normally taken to include various measures such as the provision of public information and improvements to engineering practices; however it might also include, depending on circumstances, the sensitive siting of new power lines and substations, and new homes and other buildings occupied by the public. In the UK, the Stakeholder Advisory Group on ELF EMF (SAGE) was set up to identify and explore the implications for a precautionary approach in response to concerns about possible health effects at field levels below the ICNIRP guidelines [65]. In its preliminary assessment, SAGE recommended better information for the public and optimal phasing of 132 kV overhead lines. As neither of these recommendations was likely to have a major effect on reducing exposure, a best-available "corridor option" was also identified, a moratorium on building new homes and schools in the vicinity of existing power lines, and on the construction of new power lines near to existing homes and schools. SAGE carried out a formal cost-benefit exercise which illustrated that the corridor option, whilst effective in reducing exposure, was likely to be very costly, particularly in terms of loss of land and property value.

The California EMF project [73], on the other hand, suggested that various measures within a large range of expenditures could be justified. These measures depended on the chosen policy framework; whether one starts with a utilitarian cost-benefit viewpoint or a social-justice one. In 2006, the Public Utilities Commission of the State of California affirmed a "low-cost/no-cost" policy option to mitigate EMF exposure for new utility transmission and substation projects, setting a benchmark of 4% of transmission and substation project costs as a measure of low-cost mitigation, and defining various graduated precautionary measures and the prioritisation of mitigating costs for various land use categories such as hospitals, schools, residential areas, commercial and undeveloped land [74].

The value of informing the public about precautionary measures has been called into question by studies which show that such advice may in fact heighten public concern [75-77]. Precautionary advice on mobile phone use, which was issued by the UK Department of Health following the publication of the report by the Independent Expert Group on Mobile Phones [70], has been interpreted as causing concern rather than providing reassurance [75-77]. The UK Health Protection Agency, on issuing advice on the SAGE First Interim Assessment [65] was mindful that efforts to raise awareness of possible health threats could compound anxiety, along with an attendant health detriment. This would especially be the case for people living close to existing lines, where their options to reduce exposure were limited [78]. Thus, public information should be carefully constructed to promote awareness but to avoid scare-mongering. The possible risk should not be over-stated and should be conveyed proportionately to take account of other risks to health.

The low-cost recommendation is controversial to the extent that it involves societal acceptance of the possibility of a risk that may not necessarily be fully alleviated by the proposed level of intervention. This creates an ethical dilemma for policy makers of what value should be put on a child's life. There is also a prioritisation principle, not mentioned in the EC communication, which argues against excessive expenditure on precautionary measures. Public spending on established health risks which have a large impact on society is more easily justifiable than public spending on less certain risks which have a small impact. Opportunity cost consideration also dictates that the cost of precautionary measures should be weighed alongside other possible uses of the same resources. In the case of childhood leukaemia, improving outcomes for those children who don't respond well to the current treatment regimes and research into its causes might be preferable. Alternative preventative options include the screening of newborns, and appropriate follow-up, for TEL-AML1 and other pre-disposing genetic abnormalities [79,80], although recent evidence suggests that the frequency and/or levels of the TEL-AML1 positive cells may be lower than previously reported [79,81]; or controlling levels of natural background ionising radiation, which may account for 20-30% of childhood leukaemia cases [82-84].

## Conclusions

This paper considers the application of precaution to address the possible risk of childhood leukaemia from exposure to power frequency magnetic fields. The main evidence for a risk is an epidemiological association observed in several studies and meta-analyses; however, the number of highly exposed children is small and the association could be due to a combination of selection bias, confounding and chance. Corroborating experimental evidence is limited insofar as there is no clear indication of harm at the field levels implicated; however, the aetiology of childhood leukaemia is poorly understood. Taking a precautionary approach suggests that low-cost intervention to reduce exposure is appropriate. This assumes that if the risk is real, its impact is likely to be small. It also recognises the consequential cost of any major intervention. The recommendation is controversial in that other interpretations of the data are possible, and low-cost intervention may not fully alleviate the risk. The debate shows how the EC risk management framework can be used to apply the Precautionary Principle to small and uncertain public health risks. However, despite the need for evidence-based policy making, many of the decisions remain value driven and therefore subjective.

## Competing interests

The authors declare that they have no competing interests.

## Authors' contributions

MM conceived of the evaluation. TL assessed the causes of childhood leukaemia and JS considered the epidemiological evidence. ZS and AMcK contributed to the overall discussion. All authors read and approved the revised manuscript.

## Pre-publication history

The pre-publication history for this paper can be accessed here:

http://www.biomedcentral.com/1471-2458/10/673/prepub
